# Dopamine agonist resistant prolactinomas: any alternative medical treatment?

**DOI:** 10.1007/s11102-019-00987-3

**Published:** 2019-09-14

**Authors:** P. Souteiro, N. Karavitaki

**Affiliations:** 1 Department of Endocrinology, Diabetes and Metabolism, Centro Hospitalar Universitário de São João, Porto, Portugal; 2 Faculty of Medicine of University of Porto, Porto, Portugal; 3 Instituto de Investigação e Inovação em Saúde, University of Porto, Porto, Portugal; 4 Institute of Metabolism and Systems Research (IMSR), College of Medical and Dental Sciences, University of Birmingham, IBR Tower, Level 2, Birmingham, B15 2TT UK; 5 Centre for Endocrinology, Diabetes and Metabolism, Birmingham Health Partners, Birmingham, UK; 6 Department of Endocrinology, Queen Elizabeth Hospital, University Hospitals Birmingham NHS Foundation Trust, Birmingham, UK

**Keywords:** Prolactinoma, Dopamine agonist, Resistance, Aggressive prolactinoma

## Abstract

Consensus guidelines recommend dopamine agonists (DAs) as the mainstay treatment for prolactinomas. In most patients, DAs achieve tumor shrinkage and normoprolactinemia at well tolerated doses. However, primary or, less often, secondary resistance to DAs may be also encountered representing challenging clinical scenarios. This is particularly true for aggressive prolactinomas in which surgery and radiotherapy may not achieve tumor control. In these cases, alternative medical treatments have been considered but data on their efficacy should be interpreted within the constraints of publication bias and of lack of relevant clinical trials. The limited reports on somatostatin analogues have shown conflicting results, but cases with optimal outcomes have been documented. Data on estrogen modulators and metformin are scarce and their usefulness remains to be evaluated. In many aggressive lactotroph tumors, temozolomide has demonstrated optimal outcomes, whereas for other cytotoxic agents, tyrosine kinase inhibitors and for inhibitors of mammalian target of rapamycin (mTOR), higher quality evidence is needed. Finally, promising preliminary results from in vitro and animal reports need to be further assessed and, if appropriate, translated in human studies.

## Introduction

Prolactinomas are the most common pituitary neuroendocrine tumors (PitNETs) with a prevalence ranging from 6–10 to 60 per 100,000 patients [1–3]. Most of them arise from monoclonal expansion of lactotroph cells. Less than 5% of the cases are related to a hereditary syndrome, such as Multiple Endocrine Neoplasia 1 and 4 (MEN1 and MEN4, respectively), familial isolated pituitary adenoma (FIPA) or Carney complex [4–6].

The clinical manifestations of prolactinomas relate to mass-effects (most frequently visual field defects, headaches and hypopituitarism) and/or to hyperprolactinemia-related consequences (hypogonadism and its sequelae and galactorrhea). Primary goals of treatment are reduction in tumor size, achievement of normal prolactin and restoration of gonadal function [7, 8]. The treatment of prolactinomas is unique amongst the PitNETs, since they are the only type of pituitary tumor in which first-line approach is medical therapy [with dopamine agonists (DA)] rather than surgery. Consensus guidelines recommend cabergoline in preference to other DAs, such as bromocriptine and quinagolide [7]. This is based on studies showing more optimal tolerability profile and higher efficacy in achieving normoprolactinemia and tumor shrinkage with this agent [9–12], attributed to higher affinity to D2 receptor and more potent cytocidal effect in tumor cells (compared with bromocriptine) [13]. In addition, a meta-analysis has underlined that DAs, in this case bromocriptine, can successfully manage various clinical manifestations seen in patients with prolactinoma, including 86% of those with galactorrhea, 78% with amenorrhea, 67% with sexual dysfunction, 67% with visual field defects and 53% of patients with infertility [14].

A small subset of patients does not respond to DAs (primary resistance). It should be noted, however, that definitions of resistance are highly variable throughout the literature, rendering the comparison of response rates and relevant predictors rather challenging. Practice guidelines for hyperprolactinemia suggest that a failure to achieve normal prolactin on maximally tolerated doses of DAs and a failure to achieve 50% reduction in tumor size should be regarded as DA-resistance [7]. This definition has also been acknowledged by the other publications [15, 16]. The maximally tolerated doses vary amongst patients and can be up to 12 mg weekly for cabergoline and 30 mg daily for bromocriptine [15–17]. In common clinical practice, the mean maximum dose of cabergoline (the most frequently used DA) is around 4 mg per week [18]. There is no agreement on the minimum duration of treatment and it would seem reasonable to suggest at least 6 months on the highest tolerated DA dose [17]. It is also worth mentioning that the relative importance of tumor shrinkage as a criterion for resistance needs to be challenged in adenomas in which, although reduction in size has not been achieved, they are not causing pressure effects. Consensus recommendations recognize that failure to restore fertility may also reflect treatment resistance, and that some patients might have a discordant biochemical and tumoral response, further complicating the establishment of a standard definition [7]. It should be further underlined that previous studies have used different cut-offs, such as 50% decrease in prolactin levels or 30% reduction in craniocaudal diameter of the tumor [19, 20]. Irrespective of the criteria adopted for defining DA resistance, decisions on continuing treatment with these agents should also rely on the clinical benefit (e.g. restoration of gonadal function, resolution of mass effects and particularly visual disturbances, absence of tumor growth) achieved for each individual patient.

Primary DA-resistance has been reported in approximately 20–30% of the patients on bromocriptine, and in around 10% of those on cabergoline [15, 17, 21]. Yet, when focusing on macroprolactinomas only, cabergoline fails to lead to normoprolactinemia in 17% of the cases and to tumor shrinkage in 29% of them [15]. Further studies have suggested that tumor size and invasiveness (namely cavernous sinus extension), younger age at diagnosis and male gender are predictors of lower response [18, 22–24]. Decreased expression of D2 dopamine receptors in tumor cells, alterations in other receptors modulating dopamine receptors [e.g. nerve growth factor receptor (NGFR)], changes in downstream cascades (e.g. in G protein subunit), increased angiogenic markers, and increased fibrosis through disruptions in the transforming growth factor (TGF)-β1 pathway have all been suggested as possible mechanisms playing a role in DA-resistance [25–29]. However, an extensive audit of these mechanisms is outside the scope of this review.

Secondary (or acquired) resistance to DAs is very rare and describes patients that initially responded to DA but later showed increasing prolactin levels and/or tumor enlargement. It should be pointed out that some patients who initially responded to bromocriptine but then acquired some degree of resistance have benefited from a switch to cabergoline, and, therefore, they should not be regarded as truly DA-resistant [30]. To the best of our knowledge, only six cases in the literature have reported true secondary DA-resistance, in some of them 10 years after an initial response [31–35]. The histologic characteristics of these tumors were heterogenous, ranging from adenomas without worrisome features to atypical adenomas with a high cell proliferation index. It is unknown if the mechanisms underlying secondary DA-resistance differ from those associated with primary resistance.

Current practice guidelines recommend several possible approaches for patients with DA-resistant prolactinomas [7]. In cases resistant to bromocriptine, a switch to cabergoline is recommended, based on the superior results of this agent when compared to other DAs, as previously discussed, and on studies reporting prolactin normalization in 80–85% of the patients after this change [11, 36]. Switch to quinagolide can not be excluded, although a meta-analysis found no differences when bromocriptine and quinagolide were compared for various clinical and biochemical outcomes [37]. Surgical removal is a further approach with remission rates of 63–72% and 32–60% for micro- and for non-invasive macroprolactinomas, respectively; these rates also include patients offered surgery due to DA intolerance [38, 39]. Radiotherapy is an alternative option with studies reporting normoprolactinemia rates of 15–50% that can be further increased when DA therapy is added (40–100%) [40]. Malignant and aggressive prolactinomas represent a rare and difficult setting of DA-resistance posing significant therapeutic challenges [41, 42].

The above described options to overcome DA-resistance may not always be successful and in this setting, the value of alternative medical agents has been investigated. In the following sections, we have reviewed the available literature on different pharmacological options in DA-resistant prolactinomas.

## Alternative medical treatments

### Somatostatin analogues

Somatostatin analogues (SSAs) have a well-defined role in the management algorithms of corticotropinomas, somatotropinomas and thyreotropinomas [43–45].

Immunochemistry mapping of somatostatin receptors (SSTR) has revealed that all SSTR types are present in prolactinomas; SSTR_5_ were particularly frequent, followed by SSTR_2A_ and SSTR_1_ [46–48]. However, clinical studies on the use of SSAs in prolactinomas have shown conflicting results. In the largest published case series, Sosa-Eroza et al. presented five patients with DA-resistant prolactinoma treated with octreotide LAR (20 mg for 6–13 months) in addition to cabergoline. Normoprolactinemia was not achieved in any of the patients but two of them had > 80% drop in prolactin and a > 90% reduction in tumor volume. The remaining three had no significant benefit [49]. A summary of the reported cases from the literature highlighting the mixed outcomes is shown in Table 1 [50–53].

**Table 1:** Somatostatin analogues treatment in DA-resistant prolactinomas

Study	No. of patients	Tumor	Previous treatments	SSA regime	Duration of treatment	Normal PRL (% change in PRL from baseline)	Tumor shrinkage (% volume change)
Soza-Eroza et al. [47]	5	DA-resistant macroprolactinomas	CBG (max 4.5 mg/week), surgery, TMZ, RT, tamoxifen	Octreotide LAR (20 mg/month)	12 months	No (+ 3%)	Minor (− 9%)
			CBG (max 3 mg/week)		13 months	No (+ 1%)	Minor (− 5%)
			CBG (max 3 mg/week), surgery		10 months	No (− 97%)	Yes (− 93%)
			CBG (max 4.5 mg/week), surgery		10 months	No (− 82%)	Yes (− 94%)
			CBG (max 7.5 mg/week), surgery		3 months	No (+ 5%)	Minor (− 10%)
Fusco et al. [48]	1	DA-resistant macroprolactinoma	CBG (max 3 mg/week), surgery	Octreotide LAR (20 mg/month)	NS	Yes	No
Walker et al. [49]	2	DA-resistant lactotroph carcinomas	BRC (max 30 mg/day), surgery, RT, CTX	Octreotide (100 μg 8-hourly)	NS	No	No
			BRC (max 20 mg/day), surgery, RT	NS	NS	NS	NS
Baldari et al. [50]	1	DA-resistant macroprolactinoma	BRC (max dose NS), CBG (0.5 mg/week), surgery, RT	Octreotide LAR (30 mg single dose)	Single dose	No	No
Giuffrida et al. [51]	1	DA-resistant macroprolactinoma	CBG (max dose NS), surgery, RT	Octreotide LAR (30 mg single dose)	Single dose	No	No
Coopmans et al. [55]	1	DA-resistant macroprolactinoma	BRC (max 7.5 mg/day), CBG (max 7 mg/week), surgery, RT	Lanreotide Autogel (120 mg/month)	10 months	No (+ 36%)	No
				Pasireotide LAR (60 mg/month after lanreotide was discontinued)	31 months	Yes (− 100%)	Yes (-72%)
Lasolle et al. [54]	1	DA-resistant macroprolactinoma	BRC (max 25 mg/day), QNG (max 225 μg/day), CBG (4.5 mg/week), surgery	Pasireotide LAR (initially 60 mg every 28 days, then 20 mg every 5 weeks)	7 years	Yes	No

A plus sign (+) in the PRL and volume changes indicate an increase in prolactin levels or tumor size, respectively, while a minus sign (−) imply a decrease in these variables

*SSA* somatostatin analogue, *PRL* prolactin, *DA* dopamine agonist, *CBG* cabergoline, *TMZ* temozolomide, *RT* radiotherapy, *LAR* long-acting release, *NS* not stated, *BRC* bromocriptine, *CTX* chemotherapy, *QNG* quinagolide

A theoretical advantage of the second-generation SSA pasireotide over the first-generation ones (octreotide and lanreotide) could be postulated, considering its greater affinity for SSTR_5_. Nevertheless, in vitro analyses have provided conflicting results [47, 54, 55]. The first report of a patient with prolactinoma treated with pasireotide was recently published, presenting a case not controlled after treatment with the three available DAs and undergoing two surgical procedures (Table 1) [56]. Pasireotide was then tried, achieving prolactin normalization in one month and tumor stabilization that persisted during the 7-year follow-up. No side effects were reported apart from slight deterioration of glycemic control (HbA1c increased from 5.7 to 6.2%). After this report, a second one described a DA-resistant macroprolactinoma also successfully treated with this agent [57]; considering that the tumor had a higher immunoreactivity score for SSTR_5_ than for SSTR_2_, and after attempting lanreotide Autogel without biochemical/imaging success, pasireotide was used leading to normoprolactinemia and tumor shrinkage. Once again, minor hyperglycemia was the only side effect reported (HbA1c increased from 5.4 to 6.3%).

It has been previously demonstrated that the SSTR_1_ subtype is overexpressed in DA-resistant tumors suggesting that this could be a promising therapeutic target [47]. Despite this finding, an in vitro study showed that a SSTR_1_ ligand was not highly effective in suppressing prolactin levels and the role of this receptor in prolactin secretion is still not completely understood [47].

All the aforementioned studies include a small number of patients not allowing identification of predictors of response to SSA treatment. However, it seems likely that the SSTR expression profile in lactotroph adenomas is not the only parameter associated with the high variability in the outcomes. Other less well-understood aspects of SSTR biology, such as receptor homo- and hetero- dimerization and additional downstream pathways may also play a role [58]. Further methodologically sound studies are required to clarify the place of SSAs in the treatment algorithm of DA-resistant prolactinomas. Until then, a therapeutic trial in selected patients with aggressive and DA-resistant prolactinomas could be considered as a possible option.

### Estrogen modulators

Estrogens stimulate prolactin secretion and lactotroph cell proliferation [59, 60]. Lactotroph hyperplasia leading to gland enlargement during pregnancy and breast-feeding support these findings and suggests that estrogens may be potential therapeutic targets in prolactinomas [61]. On the other hand, it is of note that prolactinomas in men are characterized by lower estrogen receptor alpha (ERα) expression which is related to higher tumor grades, resistance to treatment, and an overall worse prognosis [62].

A number of studies have evaluated the potential role of selective estrogen receptor modulators (SERMs) in prolactinoma patients (Table 2). Tamoxifen was used in 10 women previously considered bromocriptine-resistant, inducing a moderate reduction of prolactin in 6 of them [63]. Two smaller studies conducted in the pre-cabergoline era and including patients not clearly fulfilling the DA resistance criteria showed inconsistent results on the efficacy of this drug [64, 65]. Raloxifene, another SERM, resulted in minimal decrease in prolactin levels (mean reduction of 8.3 ng/mL) in 10 out of 14 patients, with the remaining ones considered as non-responders [66]. The drug was then stopped in 8 of them, as the absolute change in prolactin values was felt to be too small to justify this treatment. Fluvestrant, a selective estrogen receptor degrader (SERD) without the agonist properties of SERMs, inhibited prolactin secretion in rat prolactinoma models, but its usefulness in patients remains to be determined in clinical studies [37, 67–69].

**Table 2:** Selective estrogen receptor modulators (*SERM*) treatment in prolactinomas

Study	No. of patients	Tumor	Previous treatments	Drug regime	Duration of treatment	% Change in PRL from baseline	Tumor shrinkage (% volume change)
Volker et al. [60]	10	DA-resistant prolactinomas	BRC (median dose 5 mg/day), Surgery (n = 2)	Tamoxifen (10–20 mg/day)	4 weeks	Mean − 35% (Normal PRL in 60%)	NS
Lamberts et al. [61]	2	Prolactinomas (not DA-resistant)	NS	BRC vs. BRC/Tamofixen (20 mg/day)	36 h	45% vs. − 44%	NS
Lamberts et al. [62]	8	Invasive prolactinomas (not DA-resistant)	surgery (n = 6)	Tamoxifen (20 mg/day)	5 days	Mean − 20%	NS
Choudhary et al. [63]	14	Prolactinomas (not DA-resistant)	CGB (median dose 3 mg/week) (n = 13), BRC (15 mg/day) (n = 1)	Raloxifene (60 mg/day)	Mean 3 months	Mean − 15%	NS

A plus sign (+) in the PRL and volume changes indicate an increase in the prolactin levels or tumor size, respectively, while a minus sign (−) imply a decrease in these variables

*PRL* prolactin, *DA* dopamine agonist, *BRC* bromocriptine, *NS* not stated, *CBG* cabergoline

Aromatase inhibition blocks the conversion of testosterone to estradiol and it could possibly mitigate the estrogen-induced lactotroph proliferation [70]. A higher expression of this enzyme in prolactinomas and its correlation with tumor invasiveness has been previously shown, but this has not been confirmed in DA-resistant prolactinomas in males [71, 72]. Fadrozole administration in rats inhibited the proliferation of prolactin-positive cells and led to reduced prolactin levels [73]. Two publications have reported optimal outcomes with the use of aromatase inhibitors in DA-resistant patients with persistent hypogonadism [74, 75]. In the first case, prolactin increased after testosterone was added to cabergoline therapy. Anastrozole (1 mg daily) was then started and prolactin levels dropped by 80% in 3 months. Tumor size change was not reported [74]. In the second patient, a 36-year-old male, the introduction of testosterone replacement and human chorionic gonadotropin (hCG) therapy (in order to achieve fertility) led to raised prolactin levels. Letrozole (2.5 mg daily) was tried leading to a 74% decrease in prolactin after 32 months, improved sperm count and fertility [75]. In both cases, authors hypothesized that testosterone aromatization to estradiol and subsequent estrogen-stimulated prolactin release were the main drives for the prolactin levels increase and that aromatase inhibitors blocked this effect.

Overall, data on the use of estrogen modulators in prolactinomas not responding to DAs are limited and inconclusive.

### Metformin

Recently, metformin has attracted attention as a drug able to reduce lactotroph cells proliferation and to promote their apoptosis, both in rat xenografts and in human prolactinoma cell cultures [76, 77]. Metformin-dependent activation of AMP‐activated protein kinase (AMPK) has been proposed as the underlying mechanism in accordance with the action of metformin in other types of tumors [78]. The cascade of events downstream the AMPK activation leading to the above effects are not fully understood, but estrogen receptor downregulation seems to be involved [77].

To date, only one study has described two bromocriptine-resistant prolactinoma patients (on maximum dose of 15 mg/day) treated with metformin [79]. In the first case, a patient with prolactin levels fluctuating between 70 and 488 ng/mL was started metformin (1500 mg/day) after the diagnosis of diabetes mellitus and her prolactin was decreased to 56 ng/mL in 3 months and to 28 ng/mL in 5 months. Tumor shrinkage was also reported on both MRIs performed 5 and 10 months after drug initiation. Based on these findings, metformin was tried in a second patient of the same center. He presented with a giant prolactinoma that exhibited a good biochemical response to bromocriptine (prolactin levels decreased from 1293 to 17.7 ng/mL) but without tumor shrinkage. After starting metformin, prolactin levels decreased to 2.08 mg/dL in 3 months and a 40% reduction in tumor volume (also associated with hemorrhage) was observed.

### Temozolomide

Temozolomide (TMZ) is considered the first-line chemotherapeutic agent for aggressive pituitary tumors and carcinomas [80–82]. TMZ treatment has been reported in more than 30 lactotroph invasive adenomas/carcinomas, with approximately 50% of the patients exhibiting a decrease of more than 30% in tumor volume [83, 84].Several reports have shown dramatic improvements including disappearance of metastases, substantial primary tumor reduction and normalization of prolactin [80, 85–88]. Various regimens have been used and the administration of 50–200 mg/m^2^ for five days in 28 days cycles is the most frequently described protocol [83]. However, a second course of TMZ, even in patients previously considered as responders, has shown less favorable outcomes [89–91]. Amongst those cases, two lactotroph aggressive tumors/carcinomas were identified in which TMZ initial therapy led to good results, ranging from a remarkable 98% reduction in prolactin levels to a 25% regression in tumor size. However, when these tumors progressed and a second course of TMZ was offered, the results were disappointing, suggesting an acquired TMZ-resistance mechanism that remains to be fully clarified [89, 90].

Several studies have looked at possible predictors of TMZ response in pituitary tumors. A lower expression of O6-methylguanine-DNA methyltransferase (MGMT), a DNA-repair protein that counteracts the effects of TMZ, is significantly correlated with the effectiveness of the drug [89, 92, 93]. This observation led to the recommendation of routinely determining the MGMT status in all aggressive pituitary tumors by immunochemistry [81]. Additionally, response to TMZ in the first 3 months of treatment is considered a useful predictor of and drug discontinuation is advised if radiological progression is demonstrated after that interval [80, 81]. On the other hand, Ki-67 labelling index and p53 protein expression have not been confirmed to be of value in this setting [83, 92].

### Other cytotoxic agents

Cytotoxic agents other than TMZ have been rarely used in the treatment of aggressive pituitary tumors and carcinomas and the experience with these drugs is limited to isolated case reports. Amongst them, lomustine and 5-fluorouracil are the most frequently offered due to their ability to penetrate the central nervous system. In a series reporting four lactotroph-derived tumors (three carcinomas and one locally invasive adenoma), all of them previously surgically managed and considered DA-resistant, this combination led to a partial response only in the least aggressive one [94]. Other studies described combinations of different chemotherapeutic agents, such as procarbazine and vincristine without therapeutic success [95, 96].

### Tyrosine kinase inhibitors

The epidermal growth factor receptor (EGFR) pathway has attracted interest as a potential therapeutic target for resistant and aggressive pituitary tumors, mainly lactotroph and corticotroph ones [97]. Several receptor subtypes from this family have been identified in prolactinomas, and different expression profiles have been associated to tumor invasiveness, symptoms, and response to DAs [98, 99]. Notably, a higher expression of the ErbB3 receptor of this family in prolactinomas was associated with optic chiasm compression, suprasellar extension, carotid artery encasement, and with a better response to DA treatment [99].

Tyrosine kinase inhibitors (TKIs) block EGFR signal transduction cascades and in primary cultures of human prolactinomas, they reduce prolactin levels [100]. Two DA-resistant patients with aggressive lactotroph tumors have received treatment with lapatinib for a 6-month period. The first case achieved near normalization of prolactin and a 22% reduction in tumor volume, while the second one demonstrated a 42% reduction in prolactin levels and tumor stabilization [99]. These encouraging results are currently further explored in an ongoing phase II clinical trial in patients with DA-resistant prolactinomas [101]. Bevacizumab, a TKI targeting vascular endothelial growth factor (VEGF), has shown to partially suppress the proliferation of tumor stem-like cells isolated from rat prolactinoma [102]. This compound showed promising results in the treatment of corticotroph carcinomas but there is still no reported experience with prolactinomas [103, 104].

### Inhibitors of mammalian target of rapamycin (mTOR)

The PI3K/Akt/mTOR pathway is an intracellular signaling system regulating the cell cycle and its overactivity has been associated with several cancers [105]. Anti-proliferative responses to the inhibition of the mammalian target of rapamycin (mTOR) pathway have been reported in in vitro studies with aggressive pituitary tumors [106]. Particularly for prolactinomas, Gorvin et al. showed that certain variants of the prolactin receptor, like the Asn492Ile one, are associated with increased signaling by this pathway and cellular proliferation, and that everolimus was antagonizing these effects [107]. A case report described a patient with a DA-resistant prolactinoma that underwent multiple surgical resections and radiotherapy 6 years before a trial of everolimus (10 mg/day) was attempted [108]. After starting this agent, a 44% decrease in prolactin levels was observed and tumoral size was stable at the 1-year imaging re-evaluation. In this case, hyperglycemia, hypogeusia and mouth sores were reported as side effects. However, the same drug has been associated with disappointing outcomes in patients with aggressive corticotroph adenomas, underlining the need for larger studies [109, 110].

### Other pharmacologic agents

In addition to the aforementioned drug classes, there are others described as potentially useful in the treatment DA-resistant prolactinomas with evidence based only on pre-clinical studies.

The TGF-β1 cytokine is intimately associated with fibrotic responses in different organs and tissues. Hu et al. reported that about 43% of the DA-resistant prolactinomas were highly fibrotic and had a higher collagen content than the DA-responsive ones [79]. In addition, the expression of TGF-β1/Smad3 signaling pathway-related proteins was elevated in DA-resistant and fibrotic prolactinomas and the compound SB431542, an inhibitor of this pathway, counteracted these effects. A further publication reinforced these results, but others have reported opposite outcomes [29, 111, 112]. No studies in prolactinoma patients have been yet conducted with this drug.

A single study showed that chloroquine, an old drug used in malaria treatment, enhanced cabergoline-induced autophagy and apoptosis in prolactinoma cells in vitro [113]. The same paper also investigated two animal models in which chloroquine increased tumor suppression, allowing cabergoline to exert its effects at a lower dose. The significance of these findings in clinical practice remain to be elucidated.

## Conclusions and future perspectives

Primary or secondary resistance to DAs represent challenging clinical scenarios. This is particularly true for aggressive prolactinomas in which surgery and radiotherapy may not achieve tumor control. In these settings, alternative medical treatments have been considered but data on their efficacy should be interpreted within the constraints of publication bias and of lack of relevant clinical trials. The limited reports on SSAs have shown conflicting results, but, nonetheless, cases with optimal outcomes have been documented. Data on estrogen modulators and metformin are scarce and their usefulness remains to be evaluated. In aggressive lactotroph PitNETs, temozolomide has demonstrated optimal outcomes, whereas for other cytotoxic agents, TKIs and for mTOR inhibitors, higher quality evidence is needed. Finally, promising preliminary results from in vitro and animal reports need to be validated and translated in human studies.
